# Reversible photochromism of polynorbornenes bearing spiropyran side groups

**DOI:** 10.1007/s00706-012-0827-0

**Published:** 2012-08-29

**Authors:** Lucas Hauser, Astrid-Caroline Knall, Meinhart Roth, Gregor Trimmel, Matthias Edler, Thomas Griesser, Wolfgang Kern

**Affiliations:** 1 Institute for Chemistry and Technology of Materials, Graz University of Technology, Stremayrgasse 9, 8010 Graz, Austria; 2 Chair of Chemistry of Polymeric Materials, University of Leoben, Otto-Glöckel-Strasse 2, 8700 Leoben, Austria

**Keywords:** Photochemistry, Spiro compounds, Photolithography, Ring-opening metathesis polymerization

## Abstract

**Abstract:**

In this paper, the synthesis and characterization of poly(norbornene) homo- and copolymers bearing spiropyran side groups are described. Difficulties in the homopolymerization of spiropyrans due to the opened merocyanine form were observed leading to low polymerization yields for homopolymers while copolymers with 10 mol% spiropyran content were prepared in good yield. Spiropyrans are characterized by their reversible photochromism, which was conserved in the polymers as shown by UV–Vis spectroscopy and FT-IR spectroscopy. The switching between the apolar spiropyran form and the zwitterionic merocyanine form also leads to switchable wettability as evidenced by contact angle measurements.

**Graphical Abstract:**

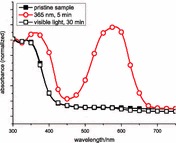

## Introduction

**Scheme 1:**
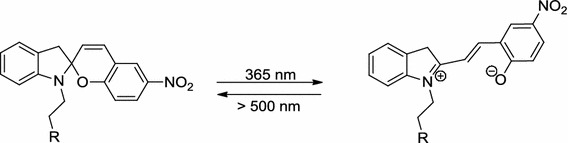

Fischer and Hirshberg [1] were the first to report the reversible photochromism of spiropyrans in 1952. Upon irradiation with ultraviolet light (365 nm), the spiro C–O bond is cleaved, resulting in the zwitterionic merocyanine form. Because of the additional double bond, an extension of the π-system in the merocyanine causes a deep blue color. A reversion of this reaction can be achieved either thermally or by irradiation with longer wavelengths (>500 nm, Scheme 1).

Due to the aforementioned zwitterionic nature of the merocyanine and the changes in their molecular conformation, the illumination of spiropyran-based photoreactive materials results in a pronounced change in their physical properties, such as wettability [2, 3], refractive index [4], or dipole moment [5]. The use of light as an external stimulus for such conversions has many advantages over thermal or electrical stimulation, like reduced chemical contamination or exact optical patterning in a nanometer range.

Together with his discovery, Hirshberg [6] already introduced the concept of an erasable optical memory in 1956, which can be seen as the starting point for intensive research in the area of organic photochromism. Berkovic et al. [7] published a comprehensive summary on the application of such compounds in photochromic liquid crystals, nonlinear optics as well as materials for real-time holography. Chemical modifications of the spiropyran ring system are easily performed allowing a huge variety of compounds to be accessed. Lukyanov et al. [8] reviewed different synthesis strategies for spiropyrans.

The photochromic switching behavior of spiropyran molecules has been previously applied in optoelectronic devices [9–13], optical data storage [14], lipid membranes [15], nanoparticles [16], hybrid inorganic/organic matrices [17], chiroptical switches [18], switchable transistors [19, 20], or DNA-binding molecules [21]. Recent endeavors focused on enhancing the persistence of the photochromism. The polarity of the microenvironment and the temperature both have a strong influence on the stability of the merocyanine form, which can be used to influence the chemical equilibrium described in Scheme 1 [22–24]. Especially the thermally induced reversion of the photomerocyanines limits the otherwise interesting application of spiropyrans in two-photon storage devices [25].

The ability of the merocyanine form to coordinate with transition metal ions further influences the equilibrium [26–29]. Natali et al. [30] observed a high selectivity of a merocyanine towards Zn(II) and explored this effect for photoswitchable sensors.

Compared to blended systems, covalent binding of functional moieties to polymer backbones has the advantage of reduced phase separation. Furthermore, the processability is improved and leaching of the functional moieties is avoided. Fries et al. [31–33] prepared methacrylate copolymers with pendant spiropyran units. Lee et al. [34] prepared a diblock copolymer with pendant spiropyran units using a PEG-functionalized ATRP initiator for reversibly photo-switchable micelles. The pronounced change in surface energy of another methacrylate copolymer due to the formed polar photomerocyanines was utilized by Higuchi et al. [35] for light-induced detachment of cells.

In this contribution, we focus on the preparation of spiropyran containing polymers via ring-opening metathesis polymerization (ROMP). Ring-opening metathesis polymerization is a versatile technique for the preparation of functional polymers [36] and is e.g., widely applied for the preparation of liquid crystalline polymers [37]. There is only limited experience on ROMP of spiro-containing monomers, e.g., in the surface initiated ROMP to obtain polymer brushes [4], because the strong coordinating nature of merocyanine is expected to have a negative impact on the polymerization performance, which will be discussed in this paper. Furthermore, the reversible photochromism will be utilized for photolithography to inscribe miniaturized structural features into films of the obtained functional (co)polymers.

## Results and discussion

### Synthesis and characterization

The spiropyran chromophore was connected to the polymerizable norbornene via a hexyl spacer and using carboxylic ester anchoring groups.

**Scheme 2:**
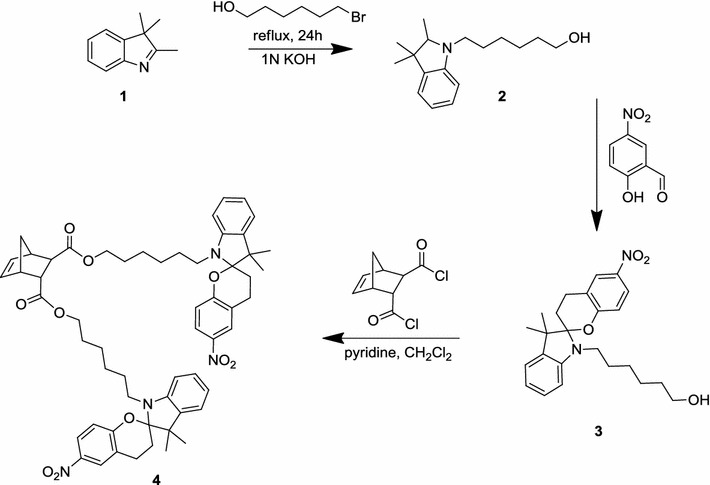

In order to increase the number of photochromic units per monomer, we targeted the difunctional (±)-*(endo*,*exo)*-bis[6-(3′,3′-dimethyl-6-nitrospiro[chromene-2,2′-indolin]-1′-yl)hexyl] bicyclo[2.2.1]hept-5-ene-2,3-dicarboxylate (**4**). In addition, the selection of the *endo*/*exo*-disubstituted norbornene rules out kinetic effects on the polymerization, which might be caused by differences in the *endo*/*exo*-selectivity of ROMP initiators [38]. Scheme 2 shows the synthesis strategy for the photochromic monomer **4**. In the first step, 2,3,3-trimethylindolenidine (**1**) was treated with 6-bromohexanol to yield 6-(2,3,3-trimethylindolin-1-yl)hexan-1-ol (**2**), which was obtained in good yield (95 %) and could be used for the next reaction step without further purification. Herein, the intermediate **2** was treated with 2-hydroxy-5-nitrobenzaldehyde leading to 1′-(6-hydroxyhexyl)-6-nitrospiro[2*H*-1-benzopyran-2,2′-indoline] [39] (**3**) with again good yield (70 % after purification). The photoreactive monomer **4** was finally prepared via esterification of **3** and (±)-*(endo*,*exo)*-bicyclo[2.2.1]hept-5-ene-2,3-dicarbonyl dichloride [40] under Schotten-Baumann (Einhorn) conditions (yield 94 %). The structure of the obtained monomer was confirmed by ^1^H, ^13^C NMR, and FT-IR spectroscopy.

### Polymerizations

**Scheme 3:**
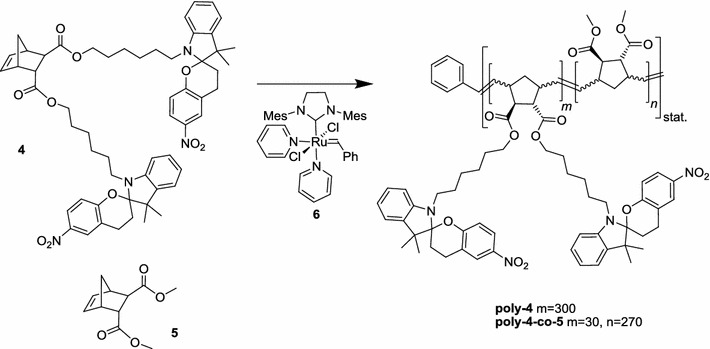

Ring-opening metathesis polymerization (ROMP) was performed in dry, degassed dichloromethane or toluene using the modified second-generation Grubbs initiator RuCl_2_(H_2_IMes)(pyridine)_2_(CHPh) (**6**) (H_2_IMes = *N*,*N*-di(mesityl)-4,5-dihydroimidazolin-2-ylidene) [41] at an overall initiator to monomer ratio of 1:300 (Scheme 3). Prior to polymerization, the monomer solution was irradiated with visible light (standard tungsten lamp) to minimize the merocyanine content.

For homopolymerization, full turnover of this reaction could not be achieved, even after 24 h of reaction time, which is usually sufficient for complete polymerization using **6** as initiator. Consequently, the polymeric products could only be obtained in low yields, ranging from 40 to 60 % (see Table 1). A strong deviation of the molar mass from the theoretically expected value, which was also accompanied by a very broad molar mass distribution was noted. The polymers were further characterized by ^1^H NMR spectroscopy (showing the typical peaks at ~5–5.6 ppm for the protons of the polynorbornene double bonds), size exclusion chromatography (SEC), and FT-IR spectroscopy.

**Table 1:** Polymerization yields and molar mass (distribution)

Polymer	Solvent	Yield/%	*M*_n_^a^/g mol^−1^	*M*_n_ (th.)/g mol^−1^	PDI	SP content	
						NMR/mol%	UV^b^/mol%
**Poly-4**	DCM	60	33,000	288,000	1.7	–	
**Poly-4**	Toluene	40	18,000	288,000	1.5	–	
**Poly-4-co-5**	DCM	66	89,700	85,700	1.3	4.0	5.3
**Poly-4-co-5**	Toluene	93	74,200	85,700	1.5	5.6	6.9

^a^Determined using size exclusion chromatography in CHCl_3_

^b^UV absorption measured at the isosbestic point for the spiropyran–merocyanine photoreaction (336 nm) in CH_2_Cl_2_ [46]

### Copolymerization of monomer **4** with (±)-(endo,exo)-bicyclo[2.2.1]hept-5-ene-2,3-dicarboxylic acid dimethyl ester (**5**)

For copolymerizations, a 30:270 molar ratio of **4**:**5** with respect to the initiator **6** was chosen. As described above, the monomer solutions were treated with visible light prior to initiation to reduce the content of the open merocyanine form in the solutions. In addition to the characterization techniques performed for the homopolymers, the content of copolymerized spiro monomers was calculated from NMR and UV–Vis spectra (see Table 1).

Comparing the results of the homopolymers with the copolymers, it is obvious that the yields as well as the polydispersity indices are not in accordance with a living type ROMP. One reason for the relatively poor polymerization results, in spite of the fact that ROMP has been successfully applied for the (co)polymerization of challenging structures [36], could be the coordinating interaction of the formed merocyanine with the Ru center resulting in a reduced number of “active” polymer chains and a non-livingness of the polymerization. Previously, similar detrimental effects caused by other coordinating ligands have been observed [42, 43]. In a recent publication dealing with the polymerization of norbornyl-derivatized spirooxazines using a G2 Grubbs initiator, similar results were observed (polymerization time 24 h, yield 66 %, PDI 1.66) which suggests a non-living polymerization [44].

Overall, polymerization yields and molar masses were higher compared to the homopolymers when copolymerization of **4** with **5** was attempted. In fact, in both solvents, the obtained molar masses were comparable to the theoretically expected result (85,700 g mol^−1^) yet with broad molar mass distribution. One possible reason for this is the lower concentration of merocyanine molecules in relation to initiator molecules. The copolymerization in toluene resulted in a better yield and a higher incorporation of spiro units in the copolymer compared to the reaction carried out in dichloromethane (Table 1). This can be explained by the fact that the open merocyanine form is less favored in apolar solvents, which also resulted in a faster discoloration of the initially bluish-colored monomer solution in toluene compared to dichloromethane upon Vis-irradiation.

### Material properties

**Fig. 1:**
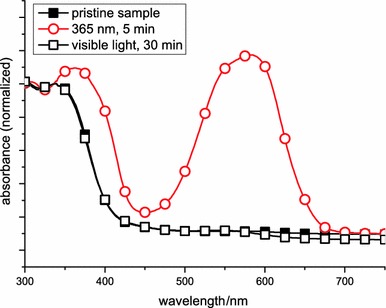
UV–Vis measurements of **poly-4** on CaF_2_ discs: before (*closed squares*), after UV illumination (365 nm, 5 min, *circles*), and after Vis illumination (60 W tungsten lamp, 30 min, *open squares*)

UV–Vis spectra and photochromic properties of the spiropyran polymer: for characterization of the reversible photochromism, thin films were spin-casted (800 rpm) from a chloroform solution (10 mg cm^−3^) of **poly-4** onto CaF_2_ discs. UV–Vis spectra before and after illumination with ultraviolet and visible light were recorded (Fig. 1).

As already known from other nitro-substituted spiropyran derivatives, the absorption spectrum of the slightly yellow spiropyran polymer shows a maximum at about 360 nm [46], which is related to the internal charge-transfer transition in the molecule. After illumination with UV light, the spiro C–O bond is cleaved, resulting in formation of the photomerocyanine. Consequently, a new maximum absorption bond at 572 nm appears and the first absorption maximum is shifted towards 380 nm. Treatment of the merocyanine form with visible light results in a reversion to the spiropyran form and in a change of the absorption maximum peaking again at 360 nm.

**Fig. 2:**
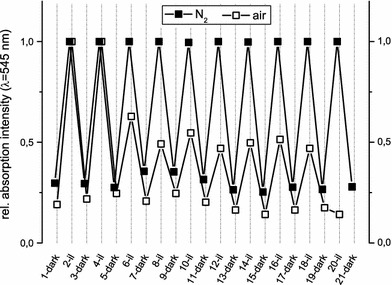
Relative UV–Vis absorption of **poly-4** after repeated cycles of UV (5 min) and Vis (30 min) illumination under ambient air (*open symbols*) and inert atmosphere (*closed symbols*)

It has to be noted that the reversibility of this process is imparted by the presence of oxygen leading to limited reversibility after three illumination cycles due to oxidation of the merocyanine form. This can be explained by the generation of singlet oxygen in presence of a long-lived triplet state in the photoisomerization process and consequent polymer degradation [44]. Under inert conditions, the photochromism was found to be reversible for more than ten UV–Vis illumination cycles (see Fig. 2).

**Fig. 3:**
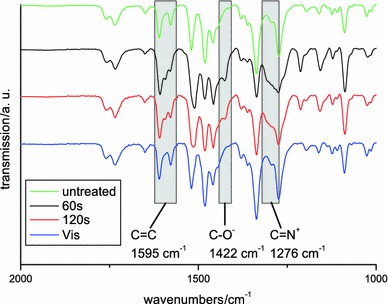
FT-IR measurement of **poly-4** before and after illumination

FT-IR measurements (Fig. 3) of irradiated samples before and after relaxation proved the ring-opening nature of the photochemical process. Figure 3 shows the according spectra. Therein, three main differences of the spiropyran and the merocyanine can be observed. After illumination broadening of the peak at 1,276 cm^−1^ is observed, which can be attributed to the building of the new C=N^+^ bond in the merocyanine form. The second evidence is a new peak showing up at 1,422 cm^−1^, which is caused by the formation of the C–O- bond in the merocyanine. Finally, a new peak is observed at 1,595 cm^−1^, which can be assigned to the C=C double bond in the center of the molecule. After illumination with visible light or thermal treatment, these peaks are again absent in the spectra of the reformed spiropyran polymers.

The zwitterionic nature of the photomerocyanine is also reflected in a pronounced change in wettability (leading to an increased hydrophilicity), which was confirmed by contact-angle measurements of **poly-4** (summarized in Table 2) using water as the test liquid. Upon irradiation and PMC formation, the contact angle changed from 106 to 92°. As already observed in UV–Vis spectroscopy, this process was reversible. For the copolymer samples (about 5–7 % of spiropyran content), however, no significant change in contact angle was observed after illumination.

**Table 2:** Contact angle/° results, test liquid water

Sample	Untreated	365/nm	572/nm	365/nm
**Poly-4**	106.0 ± 13	91.9 ± 0.5	105.4 ± 1.4	93.4 ± 1.1
**Poly-4-co-5**	89.5 ± 1.7	88.9 ± 1.2	ND	ND

### Photolithographic patterning of thin films

**Fig. 4:**
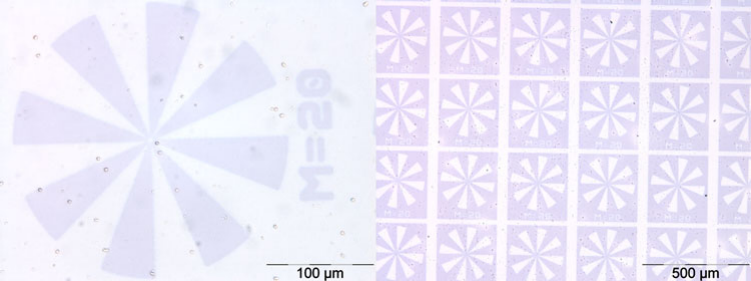
Photolithography of **poly-4** (*left*) and **poly4-co-5** (toluene, *right*) after illumination (1 min, 365 nm)

Thin films of **poly-4** and **poly4-co-5** (prepared by knife-coating from chlorobenzene solutions (10 mg cm^−3^)) were illuminated using a mask aligner system equipped with a quartz–chromium mask (contact lithography). The features of the inscribed structures were visualized by optical microscopy. In Fig. 4, photographs of patterned films are shown. Due to the deeply colored photomerocyanines, the illuminated areas are clearly visible as dark features in both polymer samples. Without any optimization, resolutions of 5 μm were achieved in this experiment. The image can be easily erased by visible light illumination or by thermal treatment. Due to the reversible nature of this photoreaction, these processes can be repeated several times.

## Conclusions

Functional monomers with pendant spiropyran groups were prepared and characterized. By ring-opening metathesis polymerization (ROMP), corresponding homopolymers and random copolymers could be obtained. While for the homopolymers only low yields and short chain lengths could be achieved, the random copolymers showed molar masses close to the theoretically expected ones.

The photoreaction of the pendant spiropyran groups is not only reflected in a pronounced change in the UV–Vis-spectrum but furthermore, surface wettability is changed. These effects were both present in the homopolymer samples. Due to the low spiropyran content, the random copolymers did not show a significant change in wettability while it was still possible to reversibly inscribe optical structures with very small features using a photolithographic technique.

## Experimental

All chemicals were purchased from commercial sources (Sigma-Aldrich, ABCR, VWR) and used without further purification, unless mentioned otherwise. Monomer **5** was obtained from Orgentis Chemicals. RuCl_2_(pyridine)(H_2_IMes)(CHPh) (**6**) was prepared according to reference [41]. 1′-(6-Hydroxyhexyl)-6-nitrospiro[2*H*-1-benzopyran-2,2′-indoline] was synthesized according to reference [39]. Solvents were distilled over appropriate drying agents (Na/K, CaH_2_) and degassed with argon or nitrogen. All experiments were carried out under inert atmosphere in a glove box or using Schlenk technique.

^1^H and ^13^C NMR spectra were recorded on a Varian INOVA 300-MHz spectrometer. Deuterated solvents were purchased from Cambridge Isotope Laboratories and solvent residual peaks were used for referencing the NMR spectra to the corresponding values given in the literature [45]. Chemical shifts are given in ppm relative to a TMS standard. Peak shape indication was done as follows: s (singlet), d (doublet), dd (doublet of doublets), t (triplet), q (quadruplet), m (multiplet), b (broad), bs (broad singlet). FT-IR spectra were recorded with a Perkin-Elmer Spectrum One instrument (spectral range between 4,000 and 450 cm^−1^). All FT-IR spectra of the samples were recorded in transmission mode (films on CaF_2_ discs). UV–Vis spectra were measured with a Shimadzu UV–VIS spectrophotometer. Contact-angle measurements were carried out with the drop shape analysis system DSA100 (Krüss GmbH, Hamburg, Germany), using water as test liquid (drop volume ~30 mm^3^). The contact angles were obtained by means of the sessile drop method and they were measured within 2 s.

Size exclusion chromatography (SEC): weight and number average molar masses (*M*_w_ and *M*_n_) as well as the polydispersity index (PDI = *M*_w_/*M*_n_) were determined by size exclusion chromatography with the following set-up: Merck Hitachi L6000 pump, separation columns from Polymer Standards Service (8 × 300 mm, STV 5 μm grade size; 106, 104, and 103 Ǻ pore size), refractive index detector (model Optilab DSP interferometric refractometer) from Wyatt Technology. Polystyrene standards from Polymer Standards Service (Mainz, Germany) were used for calibration. All SEC runs were performed using chloroform as the eluent.

UV irradiation experiments: the ring-opening UV illumination was carried out under inert atmosphere (argon) by using a 100-W ozone-free mercury low-pressure lamp (EXFO EFOS Novacure), with an irradiation time of 30 s. For ring-closure illumination, a simple tungsten lamp (60 W) was used.

Photolithographic patterning of thin films of **poly-4** and **poly-4-co-5** was performed with a mask aligner (model MJB4 from SUSS, Germany, resolution 1 μm) using a 500-W HgXe lamp equipped with a filter transmissive for the wavelength range from 270 to 353 nm for 1 min.

Caution! UV irradiation causes severe eye and skin burns. Precautions (UV protective goggles, gloves) must be taken.

### *(*±*)*-(*endo,exo*)-*Bis[6*-*(3′,3′*-*dimethyl*-*6*-*nitrospiro[chromene*-*2,2′*-*indolin]*-*1′*-*yl)hexyl] bicyclo[2.2.1]hept*-*5*-*ene*-*2,3*-*dicarboxylate* (**4**, C_57_H_62_N_4_O_10_)

6-(3′,3′-Dimethyl-6-nitrospiro[chromene-2,2′-indolin]-1′-yl)hexan-1-ol (**3**, 0.85 g, 2.1 mmol, 2.2 equiv.), which was synthesized according to the literature [39], was treated with 0.20 g bicyclo[2.2.1]hept-5-ene-2,3-dicarbonyl dichloride (0.91 mmol, one equiv.) and 0.431 g pyridine (0.440 cm^3^, 12.6 mmol, six equiv.) in absolute dichloromethane. A small amount of DMAP was added and the mixture was stirred overnight. After complete conversion, the product was extracted with HCl and NaHCO_3_ and finally dried over Na_2_SO_4_. After solvent removal, column chromatography (cyclohexane/ethyl acetate ten/one) yielded the title compound as a sticky brownish solid. Yield: 0.82 g (94 %); m.p.: amorphous, *T*_g_: not observed; ^1^H NMR (300 MHz, CDCl_3_): *δ* = 8.00 (4H, spiro^5,7^), 7.15 (2H, spiro^8^), 7.07 (2H, spiro^4′^), 6.80 (4H, spiro^5′,6′^), 6.70 (2H, spiro^7′^), 6.54 (2H, spiro^4^), 6.36, 6.25 (2H, nb^5,6^), 5.87 (2H, spiro^3^), 4.36 (4H, O–CH_2_, hexyl^1^), 3.82, 3.67 (2H, CH, nb^1,4^), 3.36 (4H, –N–CH_2_, hexyl^6^), 2.65, 2.37 (2H, CH, *endo*, *exo*, nb^2,3^), 1.95, 1.86 (2H, CH_2_, nb^7,7′^), 1.67–1.43 (8H, CH_2_, hexyl^2,5^), 1.42 (16H, CH_2_, hexyl^3^ + CH_3_), 1.17 (4H, CH_2_, hexyl^4^) ppm; ^13^C NMR (75 MHz, CDCl_3_): *δ* = 173.8, 172.6 (2C, C = O), 159.6 (2C_AR_–O, spiro^8a^), 147.0 (2C_AR_–N, spiro^7a’^), 140.8 (2C_AR_–NO_2_, spiro^6^), 137.5 (2C_AR_, spiro^3a’^), 135.9 (2C, C = C nb^5,6^), 128.0, 127.7, 125.8, 121.6, 119.3 (14C_AR_, spiro^4a,5,7,8,4′,5′,6′^), 115.4 (2C, spiro^2,2′^), 106.7 (2C_AR_, spiro^7′^), 70.8 (2CH_2_, hexyl^1^), 52.6 (2C, spiro^3^), 48.7–45.7 (4CH, 1CH_2_, nb^1,2,3,4,7^), 43.5 (2CH_2_–N, hexyl^6^), 32.4–22.6 (8CH_2_ spacer, hexyl^2,3,4,5^), 19.8 (4CH_3_) ppm; FT-IR (thin film on CaF_2_): $\bar{\nu }$ = 2,928, 2,857, 1,762, 1,728, 1,609, 1,578, 1,520, 1,481, 1,459, 1,337, 1,272, 1,179, 1,123, 1,126, 1,090 cm^−1^.

### *Poly((*±*)*-(*endo,exo*)-*bis[6*-*(3′,3′*-*dimethyl*-*6*-*nitrospiro[chromene*-*2,2′*-*indolin]*-*1′*-*yl)hexyl] bicyclo[2.2.1]hept*-*5*-*ene*-*2,3*-*dicarboxylate)* (**poly-4**)

Bis[7-(3′,3′-dimethyl-6-nitrospiro[chromene-2,2′-indolin]-1′-yl)hexyl] bicyclo[2.2.1]hept-5-ene-2,3-dicarboxylate (**4**, 500 mg, 0.5 mmol) was dissolved in 5 cm^3^ dry dichloromethane or toluene. Then, the modified second-generation Grubbs initiator RuCl_2_(H_2_IMes)(pyridine)_2_(CHPh) (**6**) [41] (300:1 ratio monomer to catalyst) was added and the reaction was stirred at room temperature for 20 h. No full turnover could be detected using TLC. The reaction was stopped by adding 0.15 cm^3^ ethyl vinyl ether. Finally the polymer was purified by subsequent precipitation steps in *n*-pentane, methanol, and again *n*-pentane and dried under vacuum. Yield: 40 % in toluene, 60 % in dichloromethane; ^1^H NMR (300 MHz, CDCl_3_): *δ* = 8.10 (spiro^5^), 7.92 (spiro^7^), 7.11–7.00 (spiro^4′,8^), 6.81–6.79 (spiro^5′6′^), 6.65–6.61 (spiro^7′^), 6.51 (spiro^4^), 5.76 (spiro^3^), 5.60–5.00 (CH of double bonds in polymer backbone), 3.97–3.95 (O–CH_2_, hexyl^1^), 3.38 (–N–CH_2_, hexyl^6^), 3.06–2.86 (cp^1,2,3,5^), 1.80 (cp^4^), 1.60–0.90 (cp^4^, hexyl^2,3,4,5^) ppm; FT-IR (thin film on CaF_2_): $\bar{\nu }$ = 2,926, 2,853, 1,760, 1,734, 1,650, 1,610, 1,578, 1,520, 1,481, 1,459, 1,379, 1,337, 1,274, 1,161, 1,123, 1,109, 1,088 cm^−1^; SEC (chloroform): *M*_n_ = 33,000 g mol^−1^, *M*_w_ = 56,400 g mol^−1^ (polymerized in DCM); *M*_n_ = 18,000 g mol^−1^, *M*_w_ = 27,300 g mol^−1^ (polymerized in toluene).

### Statistical copolymerization of **1** and (±)-(endo,exo)-bicyclo[2.2.1]hept-5-ene-2,3-dicarboxylic acid dimethyl ester (**poly-4-co-5**)

#### Polymerization in toluene

The two monomers (510 mg (2.43 mmol, 309 equiv.) of **5** and 230 mg (0.238 mmol, 30 equiv.) of **4**) were placed in a Schlenk tube and dissolved in 10 cm^3^ of absolute toluene. After degassing, the polymerization was initiated with 5.75 mg (0.00784 mmol, one equiv.) of modified second-generation Grubbs initiator (H_2_IMes) (pyridine)_2_Cl_2_Ru = CHPh (**6**). TLC (cyclohexane/ethyl acetate 5/1, KMnO_4_) after 2 h proved full turnover (the spot corresponding to the polymer exhibited a photoinduced color change). Subsequently, the polymerization was quenched with 200 mm^3^ of ethyl vinyl ether and stirred for 1 h at room temperature. Afterwards, the volume of the reaction mixture was reduced to 1 cm^3^ and the polymer was precipitated by dropwise addition of this solution to 200 cm^3^ of chilled, vigorously stirred methanol. The precipitated polymer was collected and dried in vacuo. Yield: 685 mg (93 %) of a brownish solid; ^1^H NMR (300 MHz, CDCl_3_): *δ* = 7.97 (spiro^5,7^), 7.14–7.05 (spiro^4′,8^), 6.86 (spiro^5′6′^), 6.69 (spiro^7′^), 6.52 (spiro^4^), 5.83 (spiro^3^), 5.60–5.00 (CH of double bonds in polymer backbone), 4.20–3.80 (O–CH_2_, hexyl^1^), 3.70–3.55 (CH_3_–O, dimethylester comonomer), 3.40–2.55 (–N–CH_2_, hexyl^6^, cp^1,2,3,5^), 2.20–1.75 (cp^4^), 1.65–1.20 (cp^4^, hexyl^2,3,4,5^) ppm; FT-IR (thin film on CaF_2_): $\bar{\nu }$ = 2,999, 2,953, 2,860, 1,731, 1,610, 1,579, 1,522, 1,439, 1,380, 1,339, 1,264, 1,171, 1,095 cm^−1^; SEC (chloroform): *M*_n_ = 74,200 g mol^−1^, *M*_w_ = 113,570 g mol^−1^.

#### Polymerization in dichloromethane

The two monomers (128 mg (0.606 mmol, 309 equiv.) of **5** and 56.5 mg (0.0606 mmol, 30 equiv.) of **4**) were placed in a Schlenk tube and dissolved in 10 cm^3^ of absolute dichloromethane. After degassing, the polymerization was initiated with 1.15 mg (0.0016 mmol, one equiv.) of modified second-generation Grubbs initiator RuCl_2_(H_2_IMes)(pyridine)_2_(CHPh) (**6**). TLC (cyclohexane/ethyl acetate 5/1, KMnO_4_) after 2 h proved full turnover. Subsequently, the polymerization was quenched with 200 mm^3^ of ethyl vinyl ether and stirred for 1 h at room temperature. Afterwards, the volume of the reaction mixture was reduced to 1 cm^3^ and the polymer was precipitated by dropwise addition of this solution to 200 cm^3^ of chilled, vigorously stirred methanol. The precipitated polymer was collected and dried in vacuo. Yield: 121 mg (66 %) of a slightly brownish solid; ^1^H NMR (300 MHz, CDCl_3_): *δ* = 7.97 (spiro^5,7^), 7.15–7.04 (spiro^4′,8^), 6.83 (spiro^5′6′^), 6.70 (spiro^7′^), 6.52 (spiro^4^), 5.85 (spiro^3^), 5.60–5.10 (CH of double bonds in polymer backbone), 4.20–3.80 (O–CH_2_, hexyl^1^), 3.75–3.50 (CH_3_–O, dimethylester comonomer), 3.40–2.55 (N–CH_2_, hexyl^6^, cp^1,2,3,5^), 2.15–1.70 (cp^4^), 1.65–1.10 (cp^4^, hexyl^2,3,4,5^) ppm; FT-IR (thin film on CaF_2_): $\bar{\nu }$ = 3,000, 2,951, 2,861, 2,258, 1,726, 1,610, 1,580, 1,522, 1,479, 1,438, 1,379, 1,337, 1,264, 1,197, 1,165, 1,095 cm^−1^; SEC (chloroform): *M*_n_ = 89,700 g mol^−1^, *M*_w_ = 117,700 g mol^−1^.
